# SMC-based immunity against extrachromosomal DNA elements

**DOI:** 10.1042/BST20221395

**Published:** 2023-08-16

**Authors:** Hon Wing Liu, Florian Roisné-Hamelin, Stephan Gruber

**Affiliations:** Department of Fundamental Microbiology (DMF), Faculty of Biology and Medicine (FBM), University of Lausanne (UNIL), 1015 Lausanne, Switzerland

**Keywords:** chromosome folding, defence systems, genome maintenance, Rad50, SMC complexes

## Abstract

SMC and SMC-like complexes promote chromosome folding and genome maintenance in all domains of life. Recently, they were also recognized as factors in cellular immunity against foreign DNA. In bacteria and archaea, Wadjet and Lamassu are anti-plasmid/phage defence systems, while Smc5/6 and Rad50 complexes play a role in anti-viral immunity in humans. This raises an intriguing paradox — how can the same, or closely related, complexes on one hand secure the integrity and maintenance of chromosomal DNA, while on the other recognize and restrict extrachromosomal DNA? In this minireview, we will briefly describe the latest understanding of each of these complexes in immunity including speculations on how principles of SMC(-like) function may explain how the systems recognize linear or circular forms of invading DNA.

## Introduction

Living organisms are exposed to a plethora of selfish genetic elements including viruses and transposable elements. Bacteria can actively incorporate DNA from their surroundings by various horizontal gene transfer mechanisms such as natural transformation (uptake of exogenous DNA), phage transduction or conjugation. DNA uptake can foster genetic exchange, driving rapid shifts in genome evolution that can result in enhanced fitness. However, DNA uptake can also be detrimental to the recipient cell. Undesired outcomes include acute cell damage/lysis and chronic metabolic burden due to the costs of maintenance of parasitic DNA elements [1–4]. Extrachromosomal DNA (i.e. plasmids) play a key role in the emergence and spread of resistance against antibiotics in bacteria [5]. Extrachromosomal DNA of endogenous origin can also facilitate evolution with either positive or negative consequences for the organism. Notably, DNA copy number amplification in the form of extrachromosomal DNA circles has been linked to the development of herbicide resistance in crop weed and to cancer progression in humans [6,7].

To limit the impact of potentially harmful genetic elements (often termed ‘selfish genetic elements', since they have genetic interests diverging from that of the host) [8], living organisms have developed an immune system comprising a wide arsenal of individual defences. Adaptive and innate immunity have been intensely investigated in animals. Extensive research has also been conducted on some bacterial DNA-sequence based defence systems (e.g. restriction/modification, Crispr/Cas9), as their domestication allowed the development of powerful tools in molecular biology and genetic engineering [9,10]. However, many bacterial immunity systems have only recently been discovered and are much less well understood.

Defence systems require the coordination of at least two principle molecular activities: (i) the detection of the intruder or the intrusion process by a ‘sensor'; and (ii) the execution of an ‘effector' activity, which can range from an activity directed against the invader itself to the programmed suicide of the invaded cell to preserve the cell population (‘abortive infection’, abi) [9,10]. Defence systems can mitigate entry of selfish genetic elements into the recipient cell, directly target the incoming proteins and nucleic acids or prevent efficient replication and transmission to other cells. Selfish genetic elements naturally adapt to the presence of these defences resulting in an ongoing ‘arms race' of defence and counter-defence mechanisms.

**Figure 1.**
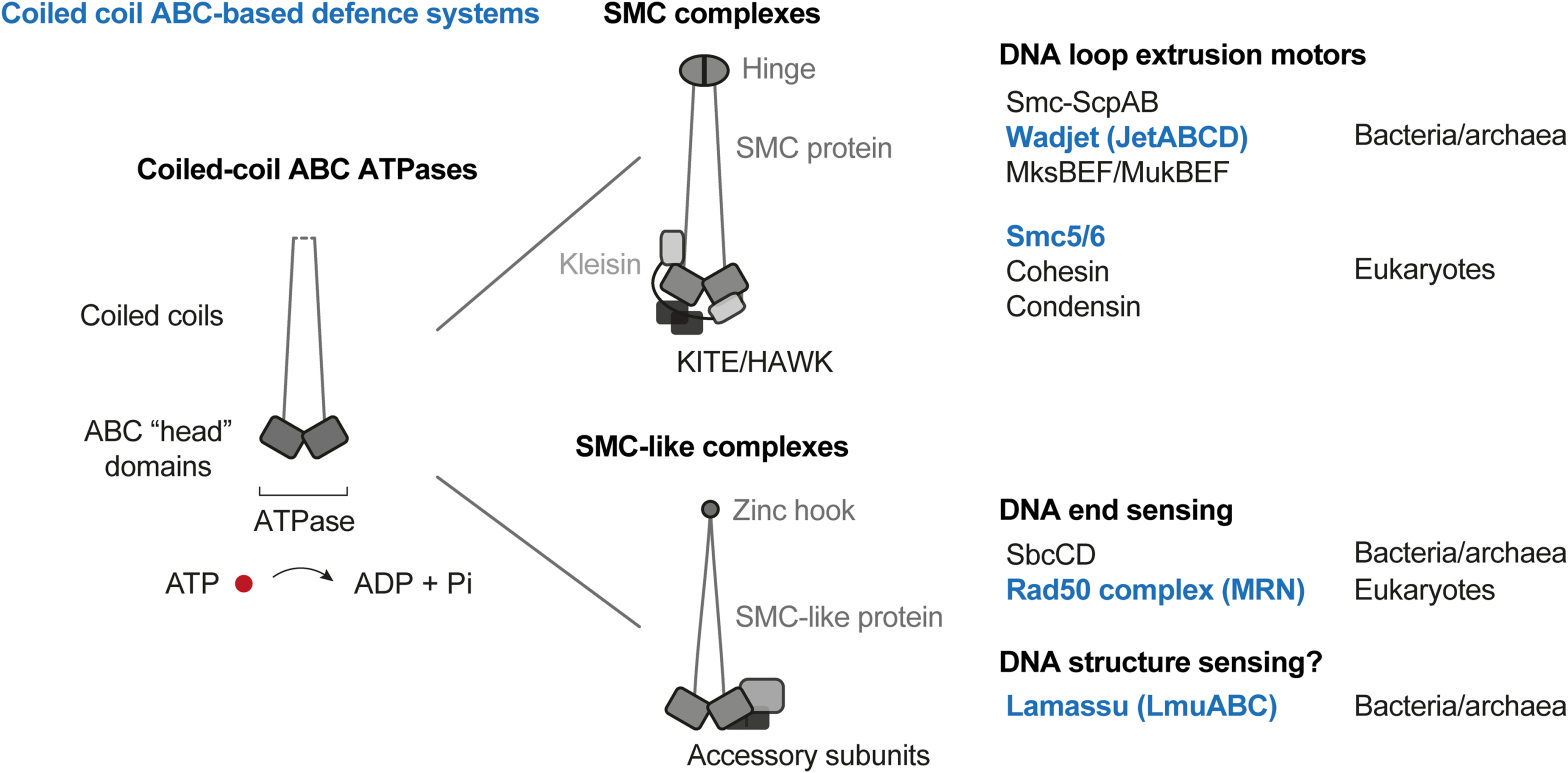
Overview of family of SMC and SMC-like complexes. Members with known roles in cellular defence are labelled in orange colours. SMC and SMC-like complexes contain proteins with ABC ATPase domains attached to long coiled-coils, ‘coiled-coil ABC ATPases'. SMC proteins harbour a hinge dimerization domain and associate with kleisin and KITE/HAWK subunits. They are thought to support DNA loop extrusion. Rad50 proteins harbour zinc hooks for dimerization and associate with the nuclease subunit Mre11. Rad50 complexes support DNA end recognition and processing.

Systematic computational searches have recently revealed a wide variety of new systems [11–13] taking advantage of their clustering in genomic locations called ‘defence islands' [14–16] or their stochastic occurrence in closely related genomes probably owing to their own genetic mobility [17]. Among the newly discovered systems, Wadjet and Lamassu are thought to act exclusively against extrachromosomal DNA. They display homology with SMC and SMC-like complexes, respectively [11], which belong to the group of so called ‘coiled-coil ABC ATPases' with roles in DNA folding and processing (Figure 1). Wadjet and Lamassu show sporadic distribution on the phylogenetic tree and high sequence divergence when compared with related coiled-coil ABC ATPases (as expected for biological conflict systems) [18]. Notably, the human Smc5/6 complex and the Rad50 complex (MRN) have independently been implicated in anti-viral immunity, underscoring the apparently broad utility of coiled-coil ABC complexes in cellular defence [19–21].

Present in all domains of life, the canonical structural maintenance of chromosomes (SMC) complexes are highly conserved master manipulators of DNA folding, critical for a myriad of processes in chromosome packaging and maintenance. SMC complexes are proteinaceous rings, comprised of two SMC proteins, containing long coiled coils that dimerize through a ‘hinge domain'. Head domains also interact to form an ABC-type ATPase. The complex is completed with a kleisin and two accessory KITE (kleisin interacting winged-helix tandem elements) or HAWK (HEAT proteins associated with kleisins) proteins (Figure 1) [22,23]. SMC complexes couple ATP binding and hydrolysis with DNA entrapment and translocation [24]. They are thought to combine these actions to fold DNA into large loops by loop extrusion [24]. ATP-dependent loop extrusion has recently been observed by isolated cohesin, condensin and Smc5/6 [25–28]. The precise way SMCs promote loop extrusion is however still in contention with several models proposed (reviewed in [24,29,30]). Eukaryotic cells typically harbour three SMC complexes: Condensin is important for mitotic chromosome packaging, cohesin for sister chromatid cohesion as well as interphase chromosome organization and DNA repair, while Smc5/6 plays a more enigmatic role in maintaining genome stability. The smaller chromosomes of bacteria are typically organized by one ‘bacterial condensin' Smc-ScpAB or MukBEF (reviewed in [24]). MukBEF is present only in enterobacteria and related species, apparently having replaced Smc-ScpAB in a common ancestor of these bacteria [31,32].

The SMC-like protein Rad50 shares the characteristic coiled-coil ABC ATPase architecture with canonical SMC proteins but does not use hinge domains for dimerization and also lacks partner proteins of the kleisin and KITE/HAWK family [33] (Figure 1). It instead uses a zinc hook for dimerization and associates with a nuclease subunit, Mre11, and (in eukaryotes) a regulatory subunit, Nbs1, to form the Rad50 complex (or MRN; also called MRX in budding yeast and SbcCD in bacteria). The Rad50 complex is a DNA end sensing and processing machine for early steps of DNA double-strand break repair and signalling. Other SMC-like proteins, RecN and RecF, are also implicated in DNA repair in bacteria likely by promoting the process of homology search during homologous recombination [32].

Below we summarize what is currently known about the roles and mechanisms of SMC-based systems in DNA immunity starting with the bacterial variants and then extending to the eukaryotic ones.

## Wadjet (JetABCD) is a bacterial SMC complex participating in anti-plasmid defence

**Figure 2.**
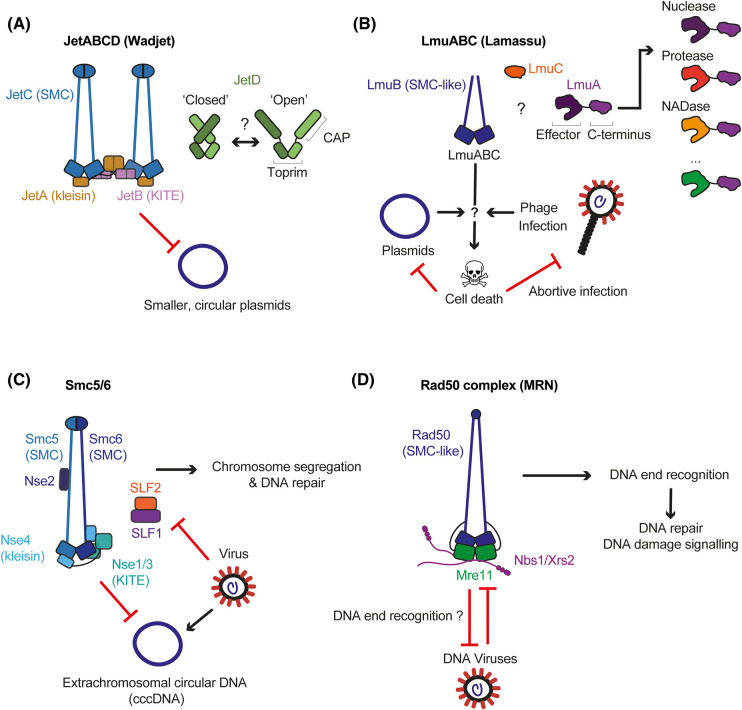
Architecture and putative activities of SMC based defence systems. (**A**) Wadjet anti-plasmid systems comprise a SMC core (JetABC, dimer of dimers) and the nuclease JetD. The JetD dimer adopts a closed inhibited conformation and presumably switches to an open active form once a plasmid has been recognized. (**B**) Lamassu (whose molecular architecture is currently unknown) is presumably formed by a dimer of the LmuB SMC-like protein associated with LmuC and LmuA proteins. The effector protein LmuA contains a domain with diverse enzymatic activities at its N-terminus [13,18,48]. Once a plasmid or a targeted phage is recognized, Lamassu may destroy the cell through its effector activity, preventing plasmid or phage spreading across the population [13,48,49]. (**C**) In eukaryotes, Smc5/6 acts as a DNA maintenance system. It can also recognize and restrict extrachromosomal circular DNA including viral genomes. Viral proteins can sequester Smc5/6 and ensure their optimal replication. (**D**) The eukaryotic Rad50 complex is a master regulator of DNA repair that specifically recognizes linear DNA ends. It can recognize and restrict some DNA viruses with such DNA ends. Viral proteins also target and inhibit Rad50 to enhance viral replication.

The Wadjet SMC complex (JetABCD) was first implicated in plasmid defence when a laboratory workhorse *Mycobacterium smegmatis* strain — inadvertently exploited for its greatly improved plasmid uptake — was found to harbour a null mutation in EptABCD (for efficient plasmid transformation; also called Muk-like SMC MksBEFG; here denoted as JetABCD) [34]. JetABCD garnered more attention in 2018 when it was caught in a computational genome sequence search for novel defence systems and widely recognized in bacterial and archaeal genomes (in ∼5–10% of bacteria) [11]. It was designated as ‘Wadjet' system after the God protector of Ancient Egypt. The additional subunit JetD was postulated to represent the anti-plasmid effector component of the complex, while JetABC is the sensor. JetA/B/C correspond to the kleisin, KITE, and SMC subunits, respectively (Figure 2A), while JetD contains a Toprim (topoisomerase-primase) domain [35,36]. All four subunits are required to suppress plasmid transformation in *B. subtilis*; according to sequence similarity and operon organization, Wadjet operons were classified into three types (I, II, and III) [11].

### JetABCD restricts smaller circular plasmids by DNA cleavage

The anti-plasmid activity of Wadjet has been observed in several instances using native and artificial test plasmids by recombinantly expressing JetABCD in *E. coli* or *B. subtilis* [11,37,38]. Studies of an endogenous system are so far limited to the instance of *C. glutamicum*, however, using non-native test plasmids [39,40]. In some cases, plasmid restriction is efficient even under growth conditions selecting for the presence of the plasmid (by antibiotic resistance). In other cases, restriction is only observed without selection. Defining the nature of plasmids targeted by Wadjet *in vivo* permitted a first hint to its mechanism. Plasmids (or extrachromosomal DNA circles) larger than ∼100 kb in size or of linear shape escape plasmid restriction [37] implying that Wadjet specifically recognizes smaller, circular DNA substrates.

By reconstituting the plasmid restriction reaction with purified proteins, JetABCD was confirmed to be a shape-sensing DNA endonuclease [37,38] that cleaves circular but not linear DNA in a sequence-independent manner. Notably, JetD alone also exhibits DNA cutting activity, but only at artificially high concentrations and lacking the specificity towards circular DNA, implying that JetABC confers the shape- and size-sensing [40]. Consistent with this notion, DNA cleavage requires ATP as well as a functioning JetC ATPase [37,38]. Importantly, DNA helical topology (and thus DNA topological stress) is not a determinant for restriction as nicked and relaxed DNA circles are efficiently cleaved by JetABCD [37,38]. The above results suggest that bacteria and archaea have remarkably harnessed an SMC-based DNA shape sensor, arming it with the executor JetD to defend themselves against small and medium-sized plasmids (<50–100 kb).

### A model to explain plasmid DNA recognition by an SMC complex

**Figure 3.**
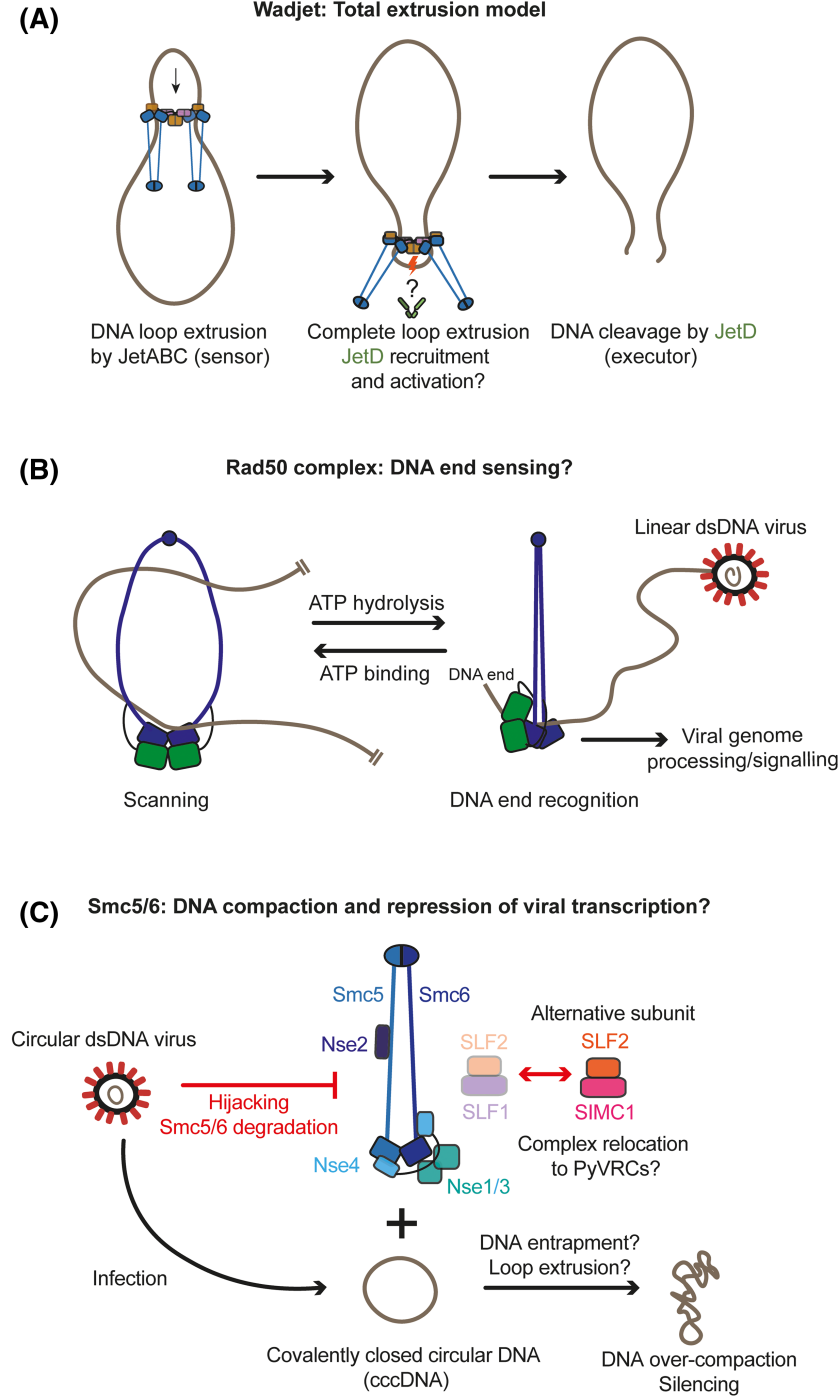
Schematics depicting the proposed defence mechanisms and pathways for each defence system. (**A**) The total loop extrusion model may explain plasmid restriction by Wadjet [37]. Both SMC motors of a Wadjet dimer-of-dimers load onto a given DNA molecule and perform loop extrusion. Completion of loop extrusion on a small circular DNA leads to self-collision of the two motors and activate DNA cleavage by JetD. (**B**) Model for DNA end capture by the Rad50 complex [69]. The Rad50 complex (Nsb1 is not represented for clarity) scans DNA to search for a DNA end. Continuous DNA (DNA loop) present in between the coiled coils prevent the coiled coils from closing. Once a DNA end is encountered, the two coiled-coil close onto a single DNA double helix leading to the formation of the cutting state, presumably facilitating various enzymatic activities (nuclease, signalling). Thus, viruses with linear DNA are expected to be targeted by the Rad50 complex. (**C**) Smc5/6, by performing DNA entrapment and loop extrusion could compact circular DNA molecules, preventing expression of viral genes that eventually harm the host cell. In some cases, a specialized loader of the complex (SIMC1/SLF2) is used for virus restriction.

Nonspecific DNA endonuclease activity would be lethal to a cell if not tightly controlled. The JetD nuclease is likely autoinhibited until relieved by JetABC action on target DNA [38,40]. Notably, the dimensions of a target plasmid are orders of magnitude larger than JetABCD itself, thus a single JetABCD complex would presumably not be able to sense the size of a DNA molecule by simply binding to it. More likely, a repeated (processive) activity is needed to detect the target's size and shape. This is in good agreement with the loop extrusion function widely associated with the canonical SMC complexes. Accordingly, JetABCD was proposed to recognize plasmid DNA by executing a loop extrusion type of activity prior to plasmid cleavage [37,38]. One specific proposition, termed the ‘total extrusion model', is that the two motor units (of a given dimeric JetABCD complex; ‘dimer-of-dimers’) that translocate in opposite directions on DNA would encounter each other in a head-to-head configuration (‘self-collision’) once loop extrusion is completed. The converging motor units may activate the JetD nuclease function [37,38]. JetABC would be dislodged from DNA ends thus explaining how linear plasmids evade restriction, while the much larger chromosomal DNA may evade restriction for example by a finite processivity of DNA translocation (a timer mechanism) (Figure 3A) or by disruption of Wadjet activity upon encounters of other Wadjet complexes, which are more likely to happen on larger DNA [37].

The total extrusion model (in its simplest form) implies that JetABC is active as loop extruder also on the chromosome. However, such activity has not yet been documented for a JetABCD system, while a (MukBEF-like) MksBEF complex in *P. aeruginosa* (which likely originated from JetABCD by loss of JetD; see below) clearly contributes to chromosome organization and segregation [35,41]. Intriguingly, Wadjet components localize to the cell poles in *C. glutamicum* [39,40], which may limit their interaction with chromosomes while at the same time facilitating plasmid encounters or promoting plasmid sequestration. While the total extrusion model provides a basic explanation for DNA recognition, plasmid restriction thus likely involves additional layers of regulation *in vivo*.

### JetABCD architecture and evolution

Recent Cryo-EM imaging has provided initial insights into how plasmid restriction might be achieved at the molecular level. Two structures of a type I JetD homodimer (of *Pseudomonas aeruginosa PA14* and *Corynebacterium glutamicum* origin) have been determined in distinct conformations. JetD was shown to comprise two domains: an N-terminal CAP (a DNA binding domain first described in the catabolite activator protein) and a C-terminal Toprim domain [11,37,38,40]. The two conformations likely represent the active and the autoinhibited form [38,40]. The JetD Toprim displays clear structural resemblance with that found in other proteins (e.g. archaeal topoisomerase VI-A, OLD-family nucleases) and mediates dimerization, as in topoisomerases. Conserved acidic residues that in topoisomerase VI coordinate a magnesium ion for DNA binding and cleavage are also present in JetD Toprim [38,40,42–45]. The JetD CAP is an elongated domain connected to the Toprim by an at least somewhat flexible linker. Despite bearing similarity with the Y-CAP domains of Topoisomerases, the JetD CAP does not contain the catalytic tyrosine that in topoisomerases support the reversible cleavage of a DNA duplex. In one structure, the CAP domains have been found closely associated with one another and presumably obstructing the putative DNA binding cleft formed by the Toprim dimer. This conformation likely represents an autoinhibited state of the enzyme [38]. In contrast, another structure showed the CAP domains pointing away from each other and from the Toprim dimer at the centre, presenting a possible active state of JetD (Figure 2A) [40]. Based on cross-linking mass spectrometry, JetD establishes multiple contacts with JetABC, including with an IFDR motif found at the N-terminus of JetB [38]. The mechanisms of JetD activation by JetABC via its transformation from the autoinhibited to the active conformation however remain to be elucidated.

Structures of two related type I JetABC cores have also been determined (*Escherichia coli GF4-3* and *Pseudomonas aeruginosa PA14*) [37,38]. Individual JetABC subunits closely resemble those of other SMC complexes, especially of MukBEF. As in MukBEF, JetABC forms a dimer-of-dimers where two SMC motors are linked together through dimerization of the kleisin N-terminus. Curiously, however, the dimer geometries differ by how the two SMC motors are positioned relative to each other [37,38]. In the MukBEF dimer-of-dimers, both motors are oriented in the same direction and are associated together in an extended I-shaped geometry [46]. In contrast, Wadjet adopts a V-shaped ‘collapsed' conformation at least in the available structures, where the two motors units are oriented in opposite directions. In a DNA-bound structure of the JetABC motor, the DNA is clamped between the coiled-coils and the ATPase, but the KITE dimer is not involved in DNA binding and rather lies on the side of the SMC head [38]. The significance of these Wadjet conformations in the process of plasmid recognition and cleavage will require further investigation.

The origin of Wadjet and the evolutionary relationship to other SMC complexes are not resolved. However, since Wadjet systems share structural features with Smc-ScpAB as well as with MukBEF, it is tempting to speculate that it emerged from an ancestral Smc-ScpAB and later gave rise to MukBEF (and likely also to MksBEF) [37,38,46]. A scenario with MukBEF and MksBEF having a history as defence system also provides a simple explanation for their highly diverged protein sequences when compared with Smc-ScpAB (and the eukaryotic SMC complexes). If so, then these defence systems have emerged from a chromosome segregation system and later gave rise to new ones [31]. Are there ways for plasmids to evade Wadjet? The answer is almost certainly ‘yes’. Wadjet operons appear to coexist with (or even be encoded in) circular plasmids (of variable sizes) in certain bacterial genomes [11]. We note that most of the research so far has been performed on type I Wadjet systems, and characterizing type II/III complexes will shed light on any mechanistic differences. Conceivably, these systems may recognize different sets of substrates and/or be resistant to distinct evasion mechanisms.

## Lamassu is a Rad50/SMC-like defence system in prokaryotes

The Lamassu superfamily (named after a protector God of Assyria) includes systems described as Lamassu type I [11] and type II [47], Lamassu-like systems [13], ABC-three components ‘ABC-3C' [18] and the DdmABC ‘DNA defence module' [48,49]. It is a large and diverse family of defence systems, widespread across all major bacterial lineages and also present in archaea (in ∼10% of the bacterial and archaeal genomes) [13]. All Lamassu systems share the SMC-like protein LmuB, which is likely the sensor component. However, unlike Wadjet, this is combined with a variety of putative effector proteins including predicted nuclease and protease subunits [13,18]. In contrast with Wadjet which targets plasmid DNA directly, Lamassu systems are thought to act through abortive infection (Abi), harming or killing the infected host to protect the cell population from invasive plasmids and phages [13,48,49].

The best studied Lamassu system (also called DdmABC) has been identified in seventh pandemic El Tor clade pathogenic *Vibrio cholerae* (7PET) that is responsible for the recent outbreaks of cholera [48,49]. Lamassu contributes to plasmid as well as phage restriction [48,49,50]. *V. cholerae* Lamassu protects cells from infection by vibriophages VIB04, VIB05, and M1Φ, [49,50], and from the λ and P1 phages when expressed in *E. coli* [48]. Various other Lamassu systems also show an anti-phage activity when recombinantly expressed in *E. coli* or *B. subtilis* [11,13,47]. At least under endogenous expression levels in the *V. cholerae* 7PET strain, Lamassu requires the presence of another defence system, DdmDE, for efficient plasmid [48] and phage restriction [50]. Plasmid-containing cells have been found to exhibit high mortality especially when plated at high cell density (a phenomenon called ‘cell density cell death'), suggesting that Lamassu activity is under control of quorum sensing [49,50]. When overproduced in *V. cholerae*, Lamassu exhibits strong plasmid-dependent toxicity — indicating that under these conditions its activation is controlled mainly by the presence of target DNA (not quorum sensing) — resulting in cells with abnormal morphologies with degraded nucleoids as well as anucleate cells. Similar, but less severe, plasmid-dependent toxicity was also observed in *E. coli.* Plasmid size does not seem to play a key role in recognition, unlike in the Wadjet systems [48].

### Composition of Lamassu systems

Lamassu systems usually comprise three components that presumably form a ternary complex: the putative effector protein LmuA, the small protein LmuC (also called middle component ‘MC' owing to its position in the operon) which is lacking in some systems, and the SMC-like protein LmuB [11,48,49] (Figure 2B). All Lamassu components, including the putative ATP binding activity of LmuB and the enzymatic activities of LmuA, are essential for the plasmid restriction and anti-phage activity of the system wherever tested [13,48,49].

The LmuA effector components harbour variable C-terminal domains, which possibly act as a docking station to the other components of the system [18]. The LmuA N-terminal domain likely represents the effector with one of various putative enzymatic activities (e.g. REase fold domain, endonuclease domains from PD-(D/E)XK and Mrr families, HNH superfamily nuclease domain, trypsin peptidase domain, C-N hydrolase, alpha /beta hydrolase, TIR domain, sirtuin (SIR2) domain, double-Rossmann dehydrogenase domain), implying that Lamassu systems can attack a plethora of cellular targets [13,18]. Several of these effector domains can also be found in other defence systems using the Abi mechanism [13,18]. For instance, the Cap4 dsDNA endonuclease domain is found in CBASS anti-phage systems as well as in many Lamassu systems including the one identified in *V. cholerae* [48,49].

LmuB is a putative relative of the Rad50 family of proteins with many (but not all) homologues harbouring a Zinc hook motif (CPxCG or similar) presumably for dimerization [18]. LmuB is relatively small (∼650 amino acids in total with relatively short coiled coils: ∼20 nm) when compared with Rad50 or SMC proteins. Generally, the C-terminal Walker B ATPase motif of LmuB carries a glutamic acid to glutamine substitution [48,49]. Equivalent substitutions engineered in diverse ABC ATPases drastically slow down the ATPase rate [51], suggesting that *Vibrio* Lamassu may exhibit low or no ATP hydrolysis activity.

### Potential substrate recognition by Lamassu

The molecular signatures that are recognized by Lamassu are not well understood. However, the similarity of LmuB to Rad50 and recent evidence indicate that LmuB may sense nucleic acid secondary structures either present in the invading species itself, or more likely formed during the infection or replication process [48,49]. The Rad50 complex specifically recognizes DNA ends (either free ends, hairpin-terminated or protein-linked). Recent lines of evidence suggest that *Vibrio* Lamassu can be activated by hairpin-forming palindromic DNA sequences contained in plasmid and phage DNA [49]. Interestingly, PriA has been found essential for Lamassu activity in *Vibrio* [49]. PriA is widely conserved across bacteria and designated as primosome assembly protein A. *E. coli* PriA has been characterized as a structure-specific DNA binding protein (fork-like hairpins, D-loops, Y structures) and 3′–5′ helicase that is a key player in the restart of stalled or abandoned replication forks. PriA is also important for the replication of the (single stranded circular DNA) phage ϕX174 as well as ColE1 plasmids, that utilize blocked fork-like DNA structures to prime their own replication [52,53]. How PriA contributes to Lamassu restriction activity is not clear. PriA may convert hairpin DNA to a substrate that is recognized by LmuABC. Alternatively, it might play a more direct role, by targeting Lamassu to phage and plasmid DNA, or helping in its activation. Importantly, PriA is essential in many bacteria, thus likely preventing evasion mechanisms based on simple PriA abrogation. Of note, however, PriA is not required for the vibriophage VIB04 infection cycle despite VIB04 being a Lamassu target [49].

Once a substrate is recognized, Lamassu would activate its LmuA effector that then attacks a wide variety of cellular targets [e.g. nucleic acids, proteins, membranes, or NAD+ (Nicotinamide adenine dinucleotide) depending on the type of effector domain] without discrimination between the host and the invader agent, leading to the destruction of the infected cell [13,18,48,49]. How the enzymatic activities of the various LmuA proteins are first autoinhibited and then activated by LmuB and LmuC is not yet clear. The Cap4 nuclease of the CBASS systems is thought to be activated by second-messenger mediated oligomerisation [54,55]. Whether related mechanisms occur in Lamassu (with a Cap4 nuclease domain) remains to be determined [48,49]. Since LmuB is putatively defective in ATP hydrolysis (see above), Lamassu activation is likely irreversible. Cooperativity during activation may be critical to avoid premature/stochastic activation.

## Smc5/6 — an antiviral SMC complex targeting extrachromosomal DNA in eukaryotes

Smc5/6 performs multifaceted roles in maintaining genome stability by acting in DNA repair and by preventing the accumulation of deleterious DNA structures. It is composed of a heterodimer of SMC proteins Smc5 and Smc6, which assembles with six regulatory non-SMC-elements (NSE), in humans Nse1–4 and SLF1/2 (reviewed in [24,56], Figure 2C). Smc5/6's participation in the cell-virus arms race was first recognized in 2016, when it was identified as a host restriction factor which is targeted by the Hepatitis B virus (HBV) protein X (HBx) in cell culture. HBx interacts with and invites the host DDB1-containing E3 ligase to mark Smc5/6 for degradation by ubiquitylation [57–59]. This mechanism of hijacking of a host E3 ligase is also employed by other viral proteins: Vpr from HIV [60], RTA from Kaposi's sarcoma-associated herpesvirus (KSHV) [61] and BNRF1 of Epstein–Barr virus (EBV) [62].

Restriction by Smc5/6 results in silencing of viral gene expression and replication [57,60,62–64]. The viral DNA targeted by Smc5/6 is thought to be extrachromosomal (Figure 3C): unintegrated elements of HIV [60], the circular episome of KSHV [61], and covalently closed circular DNA (cccDNA) of HBV [57,58]. Like in Wadjet, the restriction of extrachromosomal DNA appears to be sequence-independent and limited to circular DNA molecules [63,65]. In contrast with Wadjet, however, DNA helical topology has been suggested to play a crucial role. Accordingly, human Smc5/6 is proposed to recognize positively supercoiled DNA generated during transcription of extrachromosomal circular DNA [65]. How such a topological signal is limited to extrachromosomal DNA however remains enigmatic. Restriction of polyomavirus DNA depends on an alternative Smc5/6 loader complex (SIMC1/SLF2; instead of the canonical SLF1/SLF2). SIMC1/SLF2 moreover directs Smc5/6 to polyomavirus replication centres (PyVRCs) at promyelocytic leukaemia nuclear bodies (PML-NBs) [66]. It is unclear how two recently characterized Smc5/6 activities, DNA entrapment and loop extrusion [28,67,68], would be related to DNA topology sensing. Moreover, viral DNA (HIV and KSHV) has been characterized as hyper-compacted but whether this is a cause for or consequence of Smc5/6 action is less clear [60,61].

## The Rad50 complex, a master regulator of DNA repair involved in immunity

The Rad50 complex has also been linked to viral restriction. It is formed by the SMC-like subunit Rad50 and the endo/exonuclease Mre11. The Rad50 complex is a nuclease that specifically recognizes and processes various forms of DNA ends to initiate DNA repair by homologous recombination [69,70]. In eukaryotes, the complex together with the additional subunit Nbs1/Xrs2 has additional functions. It acts as a DNA damage signalling platform and also facilitates DNA repair via non-homologous end joining (NHEJ) (Figure 2D) [33,71,72]. At telomeres, the activity of the Rad50 complex is strictly regulated to avoid uncontrolled checkpoint activation and telomere processing resulting in telomere-telomere fusion by NHEJ [73–77].

Multiple findings implicate the Rad50 complex in host-virus interactions (Figure 2D). Similar to Smc5/6, the Rad50 complex is targeted by viruses, particularly by adenoviruses, a family of viruses with linear dsDNA genomes implicated in various human diseases. The Rad50 complex is inhibited by at least two mechanisms by adenoviral proteins, depending on viral serotype [19,20,78]: (i) the degradation of Rad50 components via a viral-cellular hybrid E3 ubiquitin ligase complex [79,80], (ii) an alteration of its location, preventing viral genome targeting by the mis-localized Rad50 complex [79,81–85]. The Rad50 complex inhibits adenoviruses likely by activating a local DNA damage response and by promoting the formation of viral genome concatemers (head-to-tail tandem repeats) by NHEJ, that are too large to be encapsulated [19,20,79,86]. Other viruses such as KSHV and HSV-1 also interact with the Rad50 complex, likely to promote their replication [87–91]. The replication of AAV viruses (adeno-associated virus and derived recombinant vectors, rAAV) appears improved in the absence of the Rad50 complex [92–95]. The Rad50 complex may even play a broader role in mammalian immunity by acting as a sensor of cytoplasmic double-stranded DNA (a marker of cell damage) and activator of interferon production [96]. In dendritic cells, Rad50 has been suggested to trigger interleukin production upon detection of cytoplasmic DNA [97]. Future research will be crucial to fully understand the roles of the Rad50 complex in anti-viral immunity.

## Conclusion

SMC and SMC-like complexes are molecular machines specialized in DNA manipulation in multiple biological processes. A growing body of evidence shows that some of these systems are involved, if not dedicated to cellular defence against extrachromosomal DNA elements. Extrachromosomal circular DNA is recognized and targeted by SMC complexes both in eukaryotes, where Smc5/6 restrict certain viruses, and in prokaryotes, where Wadjet systems target plasmids. Loop extrusion by these putative SMC motors likely senses the circularity and size of a putative target DNA molecule. In eukaryotes, the Rad50 complex is a surveillance system for DNA ends originating from abnormal cellular physiology including viral infection. In prokaryotes, it is likely that Lamassu systems also recognize aberrant DNA structures, possibly hairpin DNA ends or their derivatives.

## Perspectives

- SMC-based complexes have widespread roles in cellular immunity. They use their DNA sensing capabilities to recognize invasive DNA for restriction.
- SMC complexes — bacterial Wadjet and human Smc5/6 — restrict plasmids and viral genomes by targeting circular extrachromosomal DNA. SMC-like complexes — bacterial Lamassu and human Rad50 — likely recognize aberrant DNA structures such as DNA ends present in plasmids or during viral infection.
- Domesticated SMC-based defence systems may help to fight the spread of drug resistance.

## Abbreviations

AAV: adeno-associated virus
ABC-3C: ABC-three component
Abi: abortive infection
AF2: Alphafold 2
ATAC-seq: Assay for transposase-accessible chromatin using sequencing
cccDNA: Covalently closed circular DNA
CLMS: Cross-linking mass spectrometry
DdmABC: DNA defence module
EBV: Epstein–Barr Virus
HAWK: HEAT proteins associated with kleisins
HBV: Hepatitis B Virus
Heat: Huntingtin/EF3/PP2A/Tor1
HSV-1: Herpes Simplex Virus 1
KITE: kleisin interacting winged-helix tandem elements
KSHV: Kaposi's sarcoma-associated herpesvirus
MOI: multiplicity of infection
NAD: Nicotinamide adenine dinucleotide
NSE: Non SMC element
OLD: Overcoming lysogenization defect
PML-NB: Promyelocytic leukaemia nuclear bodies
PyVRC: Polyomavirus replication centres
SIM: SUMO interacting motif
SMC: Structural maintenance of chromosomes
Toprim: topoisomerase-primase
